# Voluntary exercise protects against methamphetamine-induced oxidative stress in brain microvasculature and disruption of the blood–brain barrier

**DOI:** 10.1186/1750-1326-8-22

**Published:** 2013-06-24

**Authors:** Michal Toborek, Melissa J Seelbach, Cetewayo S Rashid, Ibolya E András, Lei Chen, Minseon Park, Karyn A Esser

**Affiliations:** 1 Department of Biochemistry and Molecular Biology, University of Miami School of Medicine, Gautier Bldg, Room 528, 1011 NW 15th Street, 33136, Miami, USA; 2 Department of Neurosurgery, University of Kentucky, Lexington, KY, USA; 3 Graduate Center for Nutritional Sciences, University of Kentucky, Lexington, KY, USA; 4 Department of Neuroscience, Mount Sinai School of Medicine, New York, USA; 5 Department of Physiology, University of Kentucky, Lexington, KY, USA

**Keywords:** Methamphetamine, Drug abuse, Exercise, Blood-brain, Oxidative stress, Tight junctions

## Abstract

**Background:**

There is no effective therapeutic intervention developed targeting cerebrovascular toxicity of drugs of abuse, including methamphetamine (METH). We hypothesize that exercise protects against METH-induced disruption of the blood–brain barrier (BBB) by enhancing the antioxidant capacity of cerebral microvessels and modulating caveolae-associated signaling. Mice were subjected to voluntary wheel running for 5 weeks resembling the voluntary pattern of human exercise, followed by injection with METH (10 mg/kg). The frequency, duration, and intensity of each running session were monitored for each mouse via a direct data link to a computer and the running data are analyzed by Clock lab™ Analysis software. Controls included mice sedentary that did not have access to running wheels and/or injections with saline.

**Results:**

METH induced oxidative stress in brain microvessels, resulting in up regulation of caveolae-associated NAD(P)H oxidase subunits, and phosphorylation of mitochondrial protein 66Shc. Treatment with METH disrupted also the expression and colocalization of tight junction proteins. Importantly, exercise markedly attenuated these effects and protected against METH-induced disruption of the BBB integrity.

**Conclusions:**

The obtained results indicate that exercise is an important modifiable behavioral factor that can protect against METH-induced cerebrovascular toxicity. These findings may provide new strategies in preventing the toxicity of drug of abuse.

## Background

Methamphetamine (METH) is an abused drug with over 35 million users worldwide. It produces a rapid, pleasurable rush followed by euphoria, heightened attention, and increased energy. It was estimated that 10.4 million people 12 or older (i.e., 4.3% of the population) have tried methamphetamine at some time in their lives in the US (http://www.drugabuse.gov). From 1995 to 2005, admissions for primary abuse of methamphetamine/amphetamine (METH/AMPH) increased in the US from 4 to 9%. Additional 4% of all substance abuse admissions were for secondary or tertiary METH/AMPH abuse. Indeed, 66% of primary METH/AMPH abusers reported the use of other substances, including marijuana (41%), alcohol (34%), and cocaine (10%) [1]. METH abuse is three times higher in rural areas than in large cities [2]. Most METH users are white men 18 to 25 years of age; however the highest usage rates have been found in native Hawaiians, persons of more than one race, Native Americans, and men who have sex with men. Treatment of METH abuse includes cognitive behavior therapy and contingency management, although relapse rates remain high for chronic METH abusers [3].

METH abuse results in a long-term impairment of vascular functions that remain compromised even in abstinent METH users [4]. Several toxic effects of METH, such as myocardial infarction, stroke, and cardiomyopathy, are directly related to vascular or cerebrovascular dysfunction. In addition, the disruption of the blood–brain barrier (BBB) has been established as one of the most prominent events of METH toxicity [5-10]. The most significant METH-induced alterations of the BBB occur in the cortex, followed by the hippocampus [8]. These changes are correlated with neurodegeneration, perineuronal and perivascular edema, and expansion of the cortex [8,11]. Mechanistically, several aspects of METH-induced toxicity are linked to production of reactive oxygen species and induction of oxidative stress [7,9,10,12].

In the present study, we evaluated the effects of voluntary exercise on METH-induced cerebrovascular toxicity. Exercise is a modifiable behavioral factor which can produce several beneficial effects, including improved cardiac functions and musculoskeletal health [13]. Compelling evidence demonstrates the efficacy of exercise in reduction of morbidity associated with cardiovascular disease, obesity, and diabetes, as well as in cancer prevention. It is recognized that exercise can affect oxidative metabolism. For example, an aerobic physical activity program induced antioxidant enzyme activities, elevated resistance to oxidation of low density lipoproteins (LDL), and decreased levels of oxidized LDL in young and elderly patients with various forms of vascular diseases [14]. In addition, plasma antioxidant levels are correlated with physical performance [15]. Exercise is also known to decrease levels of inflammatory mediators [16].

To date there is no effective therapy available to protect against METH toxicity. Therefore, the aim of the present study was to evaluate the neurobiological effects of exercise on cerebrovascular toxicity of METH and, more specifically, METH-induced disruption of the BBB and induction of neuroinflammatory responses in the brain. Our results indicate for the first time that exercise can protect against METH-induced oxidation stress in brain capillaries and disruption of the BBB by enhancing the antioxidant protection of brain microvessels. Overall, this study strongly suggests that exercise may protect against cerebrovascular toxicity of drugs of abuse, such as METH.

## Results

### Voluntary wheel running as a model of endurance exercise in mice

**Figure 1:**
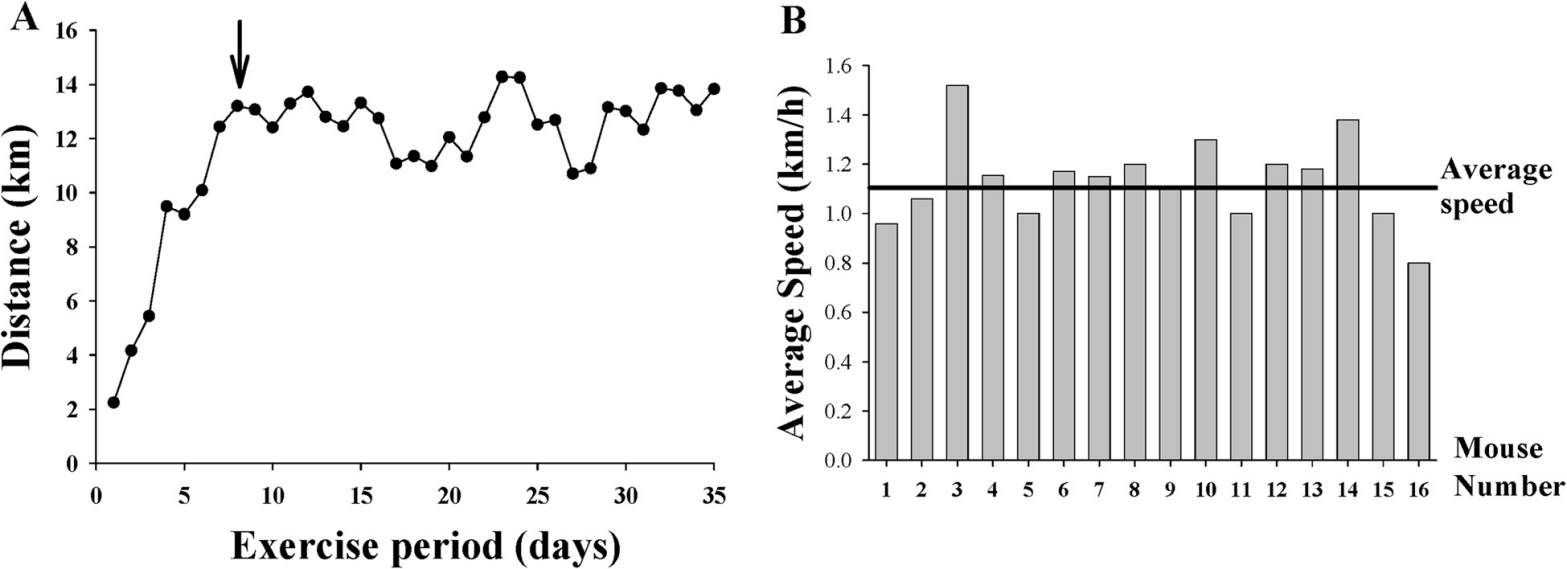
**Characterization of the voluntary wheel running.** (**A**) Typical running pattern of a mouse subjected to voluntary wheel running. The arrow indicates the end of a one week adaptation process to adjust to running behavior and solitary environment. (**B**) Average speed (km/h) for individual mice used in the present study.

A typical running pattern over a 5-week wheel running is shown in Figure 1A. As indicated, mice required one week adaptation period to the running wheel and then maintained the running activity for the duration of the study, running on average 12 to 14 km per 24 h. The mice used the running wheels regularly and ran on average for 10.3 ± 0.33 h/day. Figure 1B is a histogram illustrating individual running behavior of 16 mice that have been used in the present study. The average speed of running over a 5 week period is plotted for each mouse. The average speed for all mice was 1.12 km/h and is marked as a vertical line. Variability in the running speed ranged from 0.81 - 1.5 km/h. Endurance training employed in our study slightly decreased the body weight of studied mice (30.9 ± 0.41 g for sedentary mice vs. 28.4 ± 0.3 g for exercised mice).

### Exercise protects against METH-induced oxidative reactions in brain capillaries

**Figure 2:**
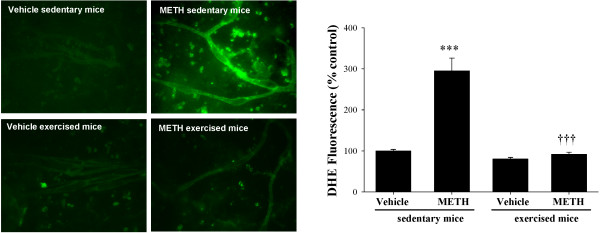
**Exercise protects against METH**-**induced oxidative stress in brain capillaries.** Mice were subjected to voluntary wheel running for 5 weeks. Control (i.e., sedentary) mice did not have access to wheels. At the end of the exercise regimen, mice were injected with METH (10 mg/kg, i.p.) for 24 h. Superoxide levels were determined in isolated brain capillaries by dihydroethidine (DHE). The images are representative data from three independent experiments and the quantified results (mean ± SEM) are depicted in the form of bar graphs. ***As compared to the respective controls at p < 0.001. †††Data in the exercised group are significantly different as compared to the respective treatment in the sedentary group at p < 0.001.

In the first series of experiments, we determined oxidative stress by DHE staining in brain capillaries from exercised and sedentary mice exposed to METH (10 mg/kg, i.p.) or saline (vehicle control) for 24 h. As indicated in Figure 2, METH exposure in sedentary mice resulted in a striking increase in DHE fluorescence; however, this effect was completely abolished in exercised mice.

**Figure 3:**
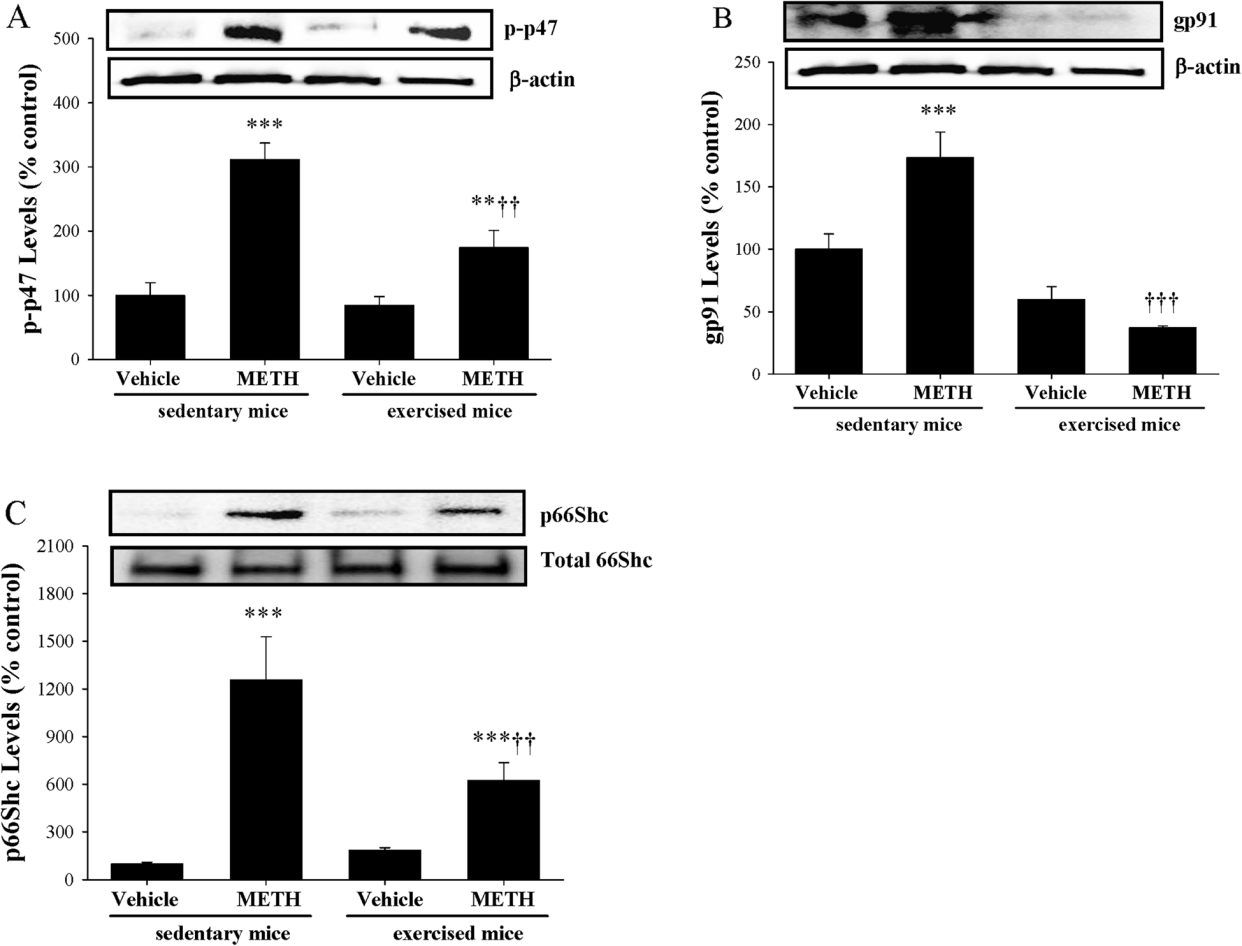
**Exercise attenuates METH**-**induced up regulation of prooxidative enzymes.** Mice were exercised and exposed to METH as described in the legend to Figure 2. Regulatory subunits of NOX, such as phosphorylated-p47 (**A**; p-p47) and gp91 (**B**), as well as phosphorylated mitochondrial protein 66Shc (p66Shc) (**C**) were determined by immunoblotting. Levels of p-47 and gp61 were normalized to β-actin and levels of p66Shc were normalized to total 66Shs. The blots are representative data from three independent experiments and the quantified results (mean ± SEM) are depicted in the form of bar graphs. *As compared to the respective controls at **p < 0.01 or ***p < 0.001. ^†^Data in the exercised group are significantly different as compared to the respective treatment in the sedentary group at ^††^p < 0.01 or ^†††^p < 0.001.

We next focused on potential mechanisms of METH-induced superoxide radical production in brain capillaries. Recent evidence indicates that NAD (P)H oxidase (NOX) is the primary prooxidative enzyme in vascular tissue. Therefore, we evaluated the effects of METH on expression of regulatory NOX subunits, such as p47 and gp91. Exposure of sedentary mice to METH resulted in increased phosphorylation of p47 (Figure 3A) and increased expression of gp91 (Figure 3B). Importantly, exercise attenuated these effects. In addition, METH treatment stimulated phosphorylation of 66Shc, a potent prooxidative enzyme localized in mitochondria (Figure 3C).

### Exercise stimulates antioxidative protection in brain capillaries

**Figure 4:**
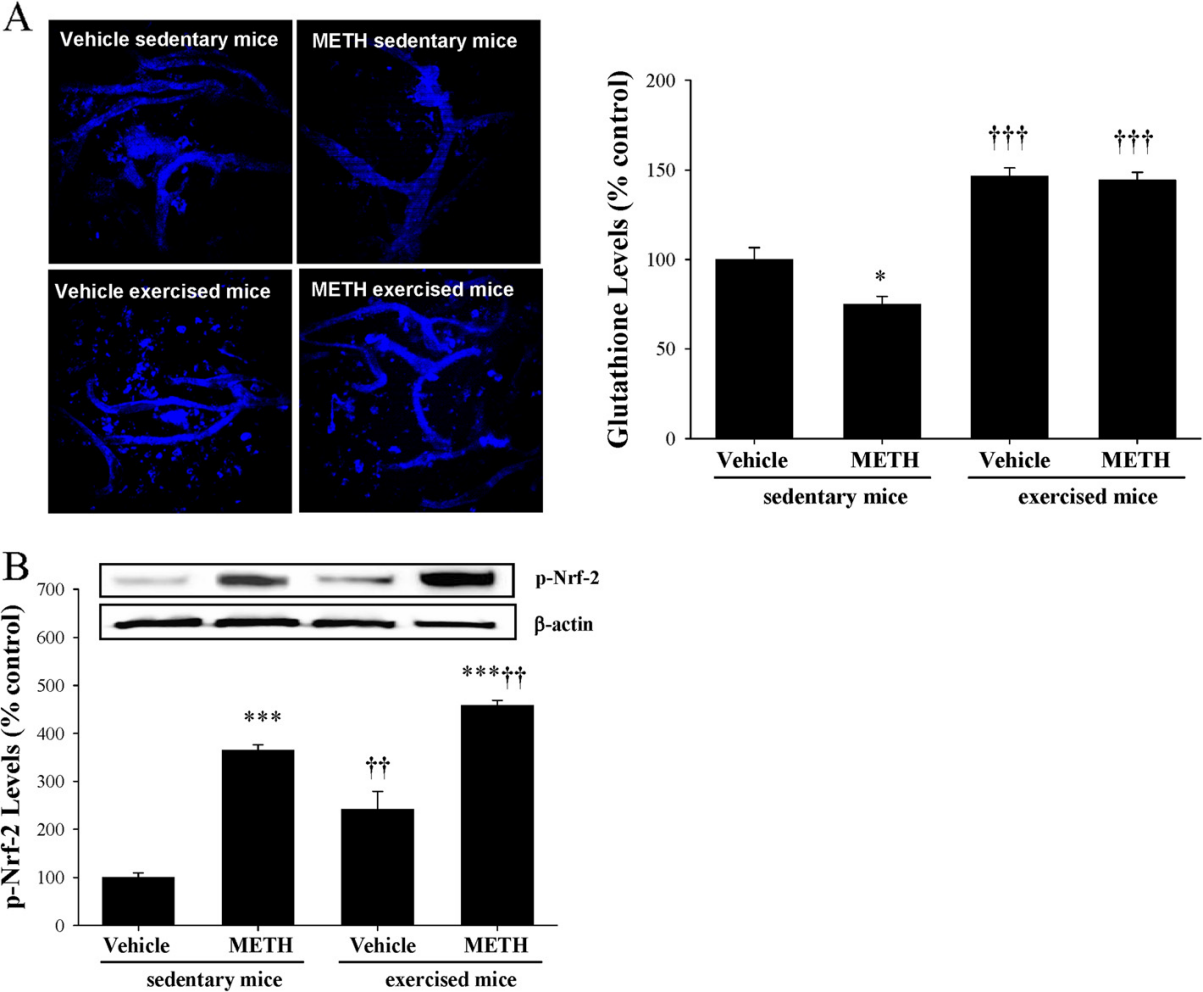
**Exercise induces antioxidant protection in brain capillaries of METH**-**exposed mice.** Mice were exercised and exposed to METH as described in the legend to Figure 2. (**A**) Glutathione levels were determined in isolated brain capillaries by staining with monochlorobimane (MCB). The images (left panel) are representative data from three independent experiments and the quantified results (mean ± SEM) are depicted in the form of the bar graph (right panel). (**B**) The expression of phosphorylated Nrf-2 (p-Nrf-2) was determined in brain capillaries by immunoblotting and normalized to β-actin level. The blots are representative data from three independent experiments and the quantified results (mean ± SEM) are depicted in the form of bar graphs. *As compared to the respective controls at *p < 0.05 or ***p < 0.001. ^†^Data in the exercised group are significantly different as compared to the respective treatment in the sedentary group at ^††^p < 0.01 or ^†††^p < 0.001.

Oxidative stress is frequently associated with changes in antioxidant levels. Therefore, we assessed the tissue levels of glutathione in exercised and sedentary mice exposed to METH. As shown in Figure 4A, exposure to METH for 3 h resulted in decreased glutathione levels in brain capillaries in sedentary animals. Exercise slightly increased the baseline levels of glutathione in brain capillaries. In addition, exercise prevented the METH-induced alterations of glutathione levels.

Activation of transcription factor Nrf-2 can provide another compensatory mechanism to protect tissues from increased oxidative stress by inducing expression of antioxidative enzymes. Figure 4B indicates that treatment with METH increased Nrf-2 phosphorylation in brain capillaries. Importantly, the expression of phosphorylated Nrf-2 was even more pronounced in brain capillaries of exercised mice exposed to METH as compared to METH-treated sedentary animals, further demonstrating the protective effects of exercise against METH-induced tissue oxidative reactions.

### Exercise protects against METH-induced disruption and redistribution of TJ proteins

**Figure 5:**
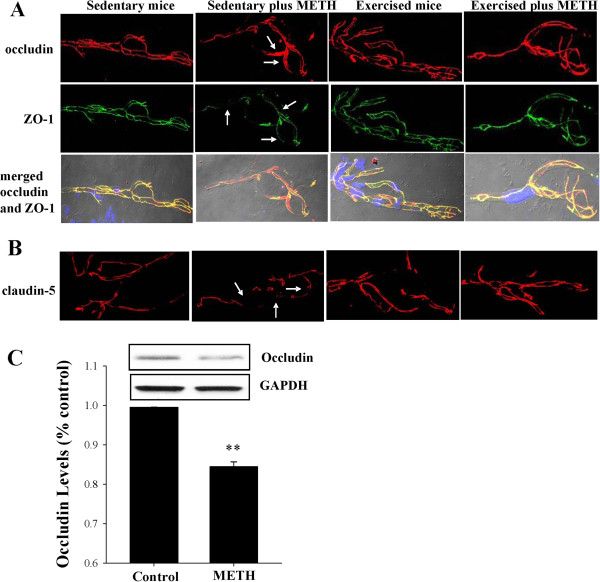
**Exercise protects against METH**-**induced fragmentation and redistribution of TJ proteins in brain capillaries.** (**A** and **B**) Mice were subjected to exercise and/or METH exposure as described in the legend to Figure 2. Top row illustrates occludin immunoreactivity, followed by ZO-1, and merged images of occludin and ZO-1 (combined with the Nomarski technique to visualize capillaries). The bottom row illustrated claudin-5 immunoreactivity. In sedentary mice, METH exposure resulted in redistribution of occludin and decreased expression and fragmentation of ZO-1 and claudin-5 (arrows). These effects were markedly attenuated in exercised mice exposed to METH. (**C**) Mice were administered with METH (10 mg/kg, i.p.) for 1 h and occludin was assessed in isolated microvessels by immunoblotting. Data are mean ± SEM, n = 4. **As compared to the respective controls at p < 0.01.

Intact TJs are the critical elements that regulate integrity and the barrier function of the brain endothelium. Disruption of TJs promotes neuroinflammatory responses by allowing paracellular entry of inflammatory cells into the brain. Therefore, we evaluated the effects of METH and/or exercise on TJ protein expression in brain microvasculature 24 h post METH treatment (Figure 5A and 5B). We focused on integrity and co-localization of TJ proteins as detected by immunofluorescence as they frequently define a proper function of the cerebral vasculature. Isolated brain capillaries were stained for transmembrane TJ proteins, occludin and claudin-5, and for TJ accessory protein ZO-1. In sedentary controls, occludin and ZO-1 staining revealed characteristic linear immunoreactivity consistent with TJ areas at the borders of adjacent endothelial cells. METH exposure in sedentary mice resulted in dramatic changes in occludin immunoreactivity that shifted from the TJ areas into endothelial cell cytoplasm (arrows in Figure 5A, the top row). Changes in occludin staining were accompanied by a decrease and fragmentation of ZO-1 immunoreactivity in METH-exposed sedentary mice (arrows in Figure 5A, middle row). As the result, occludin and ZO-1 colocalization pattern was markedly altered as illustrated in merged images that were additionally combined with Nomarski interference contrast technique to visualize the capillaries (Figure 5A, bottom row). In addition to changes in occludin and ZO-1, exposure to METH in sedentary mice resulted in decreased and fragmented immunoreactivity of claudin-5 in brain capillary (arrows in Figure 5B). However, exercise effectively attenuated these changes.

Published data reported that METH treatment can diminish TJ protein expression even at early exposure time in brain endothelial cells. This possibility was also tested in our present study. Mice were injected with METH (10 mg/kg) for 1 h, followed by estimation of occludin levels in brain capillaries. Figure 5C illustrates that such a short exposure to METH was sufficient to significantly decrease occludin level.

### Exercise attenuates METH-induced disruption of the BBB integrity

**Figure 6:**
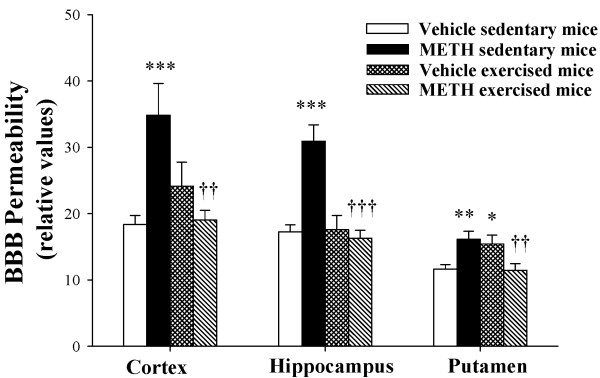
**Exercise**-**mediated protection against METH**-**induced disruption of the BBB.** Mice were exercised as described in Figure 2 and exposed to METH (10 mg/kg) for 1 h. The BBB integrity was evaluated by i.p. injecting 100 μl 2% sodium fluorescein (NaF). Fifteen minutes post NaF injection, permeability across the BBB was calculated in the discrete brain regions (hippocampus, cortex, and putament) as the ratio of fluorescence in the brain tissue to plasma. The data are mean SEM, n = 5. *As compared to the respective controls at *p < 0.05, **p < 0.01, or ***p < 0.001. ^†^Data in the exercised group are significantly different as compared to the respective treatment in the sedentary group at ^††^p < 0.01 or ^†††^p < 0.001.

Disruption of the BBB may be the ultimate outcome of METH cerebrovascular toxicity. Indeed, alterations of the BBB integrity are directly involved in trafficking of inflammatory cells into the brain and in the development of neuroinflammatory responses. Therefore, we analyzed the effects of METH and exercise on permeability across the BBB in discrete brain regions. As shown in Figure 6, BBB integrity in sedentary mice was significantly disrupted 1 h post METH administration, consistent with occludin expression changes, which occurred at the same time point. The most sensitive brain regions to METH toxicity were the cortex and the hippocampus. Importantly, exercise markedly attenuated BBB hyper permeability induced by METH in all brain regions in exercised mice. Treatment with METH (10 mg/kg) for 12 or 24 h did not result in functional changes of BBB integrity (data not shown).

## Discussion

Behavioral factors, such as physical activity, may influence substance abuse and toxicity. Exercise has been shown to activate brain reward pathways [17]; thus, it may regulate the initiation of drug abuse and modulate the development of addiction [18]. For example, studies on tobacco cessation indicated that exercise can effectively control cigarette cravings [19]. It was also reported that METH exposure can decrease proliferation and survival of medial prefrontal cortex precursors and that exercise protected against this effect [20]. However, the effects of exercise on cerebrovascular toxicity of drugs of abuse, including METH, are unknown. In the present study, we propose that enhanced antioxidant capacity of cerebral capillaries by exercise is the main mechanism responsible for exercise-mediated protection against METH-induced cerebrovascular toxicity. To address this hypothesis, we employed voluntary wheel running system that is an established model of endurance exercise for mice. The model is based on a computerized wheel cage system, allowing the mice to voluntarily run to more closely mimic human behavior. Taking into consideration the CNS effects of METH, we specifically focused our study on the BBB.

Evidence indicates that induction of oxidative stress may contribute to METH toxicity [7-10,12]. This mechanism may have particular significance in the CNS, because reactive oxygen species (ROS) are generated continuously in the brain during normal metabolism and neuronal activity to meet the high energy demand of the brain. The brain is highly sensitive to any imbalance in ROS due to it its high oxygen consumption, high iron and lipid contents, and low activity for antioxidant defences. Likewise, ROS disequilibrium in the brain is associated with several CNS disease states [21]. Our present data demonstrate that treatment with METH induced oxidative stress at the level of brain capillaries. DHE staining used in these analyses specifically indicates the prominent increase in superoxide radicals in brain capillaries of mice injected with METH. These results on METH-induced tissue oxidation in brain microvasculature are consistent with literature reports indicating stimulation of oxidative stress in brain endothelial cells upon METH exposure [9,10,12,22]. Increased levels of lipid per oxidation products were also demonstrated in chronic users of METH [22-24]. The role of oxidative stress in METH-induced toxicity is further supported by the observations that administration of antioxidants can attenuate METH-induced cellular toxicity. In addition, mice overexpressing copper-zinc or manganese superoxide dismutase (CuZnSOD or MnSOD, respectively) are protected against neurotoxicity of METH [25].

Several mechanisms, including autoxidation of dopamine and 6-hydroxydopamine and/or autoxidation of serotonin, may be responsible for METH-induced oxidation [26]. However, these factors are unlikely to play a major role in METH-induced oxidation in cells or tissues that lack dopaminergic or serotonergic innervations. Our recent data indicated the METH-induced activation of NOX is an important source of ROS in brain endothelial cells. Indeed, inhibition of NOX by NSC 23766 attenuated METH-induced ROS generation, an effect associated with protection against changes in occludin expression, and transendothelial monocyte migration [10]. The results of the present study indicate that treatment with METH can induce phosphorylation of p47 and expression levels of gp91, both being the regulatory subunits of NOX. The importance of these findings rely on the fact that phosphorylation of p47 stimulates its translocation into cell membranes and interaction with other subunits, such as gp91 to assembly of active NOX. An active NOX can induce oxidative stress cascade by generation of superoxide due to one electron transfer from NADH or NADPH [27]. Furthermore, our study indicated that METH treatment can increase the levels of p66Shc, a redox enzyme that generates reactive oxygen species in mitochondria via subtraction of electrons from the mitochondrial electron transport chain to catalyze the partial reduction of molecular oxygen [28,29].

The effects of exercise on the induction of antioxidative enzymes and protection against lipoprotein oxidation are well recognized [14-16]. Novel results of the present study indicate that exercise can provide remarkable antioxidative protection at the level of brain capillaries. We focused on the effects of exercise and/or METH on capillary glutathione because our previous studies determined that glutathione levels are an excellent indicator of oxidative stress in endothelial cells [30]. The quantitative and qualitative results of MCB staining indicate that an acute METH exposure results in a decrease in glutathione levels in brain capillaries. These results are in agreement with earlier data that glutathione is susceptible to METH treatment, as its levels are diminished in METH abusers [23]. While exercise did not alter the baseline levels of glutathione, it markedly protected against METH-induced a decrease in glutathione levels in brain capillaries.

We also evaluated the effects of METH and/or exercise on phosphorylation of Nrf-2, a transcription factor that activates antioxidant responses. Normally, Nrf-2 is repressed in cytoplasm by a protein called Kelch-like erythroid CNC homologue (ECH)-associated protein 1 (Keap1). Nrf-2 is activated and translocates into the nuclei upon phosphorylation mediated by several kinases, including those of the Ras pathway [31]. The Ras cascade is regulated by cellular redox status; thus, stimulation of Nrf-2 is consistent with METH being a prooxidative factors. Nevertheless, activation of Nrf-2 provides a mechanism of antioxidative protection by binding to the antioxidant response elements (ARE), which are present in the promoter regions of several antioxidative enzymes, including MnSOD [32]. An increase in phosphorylation of Nrf-2 by exercise is consistent with antioxidative effects of physical activity.

Intercellular junctions are involved in the regulation of integrity of the brain endothelium and the BBB functions [33-35]. TJs allow for very close contact between adjacent endothelial cells. In addition, cytosolic proteins, e.g., ZO-1 (zonula occludens-1) and ZO-2 are associated with the cytoplasmic surface of TJs. These proteins serve as recognition proteins for TJ placement and act as support structures for signaling proteins. Occludin contributes to the electrical barrier, fence and signaling functions of TJs [33-35]. Occludin is also a target to METH-induced changes in endothelial barrier function. Importantly, a METH-induced decrease in occludin levels is an early event, occurring within 1 h following METH treatment as demonstrated in both cultured brain endothelial cells [10] and brain capillaries in the current manuscript. Such a rapid response may explain early changes in BBB permeability, which were also observed in the present study. On the other hand, changes in body temperature, which occur following METH exposure, have also been linked to increased BBB permeability [36]. Disruption of the BBB may directly contribute to METH neurotoxicity and neuroinflammatory responses by allowing blood-born inflammatory cells entry into the brain. Therefore, it is important that exercise prevented METH-induced BBB breakdown. In addition to occludin, METH exposure decreased expression of claudin-5, a TJ protein that was specifically linked to diminished endothelial barrier function and disruption of the BBB [37]. In fact, knockout of claudin-5 resulted in a selective increase in paracellular permeability of small molecules across the BBB [38]. Thus, exercise-mediated protection against changes in claudin-5 immunoreactivity provides another important mechanism of protection against METH-induced alteration of the BBB integrity.

METH-induced changes in expression and immunoreactivity of TJ proteins may be caused by induction of oxidative stress. For example, studies performed in our and other laboratories demonstrated that oxidative stress can alter the integrity of the BBB at the level of TJs acting through Ras and/or Rho redox responsive signaling [39-41]. These pathways were demonstrated to play important roles in the regulation of claudin-5, ZO-1, and ZO-2 expression as well as the BBB assembly [39-43]. Other pathways that have been shown to affect TJ proteins are also redox-responsive and include the MAP kinase cascade, STAT1, and PI3 kinase.

## Conclusions

Our results indicate that acute exposure to METH induces profound oxidative stress and disruption of TJ proteins in cerebral microvessels. Most importantly, endurance exercise training protects against these effects and prevents METH-induced disruption of the BBB integrity. This data is important for a better understanding of the molecular mechanisms underlying METH-related cerebrovascular injury and indicates that exercise can protect against the cerebrovascular component of METH-induced neurotoxicity.

## Materials and methods

### Animals, experimental groups, and isolation of brain capillaries

The study was performed on male C57BL/6 mice (7–8 weeks old; weight 25–28 g, Harlan Laboratories, Indianapolis, IN). C57BL/6 mice are prone to develop addiction [17] and exert high running behavior. Mice were divided into the exercise and sedentary groups. The exercise group was subjected to voluntary running in modified shoebox wheel cages (Coulbourn Instruments, Whitehall, PA). The frequency, duration, and intensity of each running session were monitored for each mouse via a direct data link to a computer and the running data are analyzed by Clock lab and Mat lab software (Actimetrics, Wilmette, IL, and Natick, MA, respectively). Mice exercised for 5 weeks, including one week of adaptation period during which mice adjusted to solitary living and running wheel. The sedentary (control) group of mice did not have access to running wheels. At the end of 5 weeks exercise period, both the exercised and sedentary mice were injected i.p. either with a single dose of METH (10 mg/kg as D-methamphetamine hydrochloride; Sigma) or with vehicle (saline). An acute exposure to one dose of METH is relevant to individuals who initiate METH abuse, which occur frequently in young age. A single dose of METH was shown to increase attention, concentration, and psychomotor performance, which may result in developing the drug dependency [44]. The dose of METH used in this study was consistent with doses used by abusers who may use this drug in the amounts as high as 1 g/day [45].

The majority of the experiments were terminated and the animals were perfused with saline 24 h post METH injection. The BBB is formed at the level of cerebral microvessels; therefore, brain capillaries were used as the main experimental material in this study. Capillary-enriched fraction was isolated from brains as described earlier [46]. Briefly, mice were euthanized; brains were removed and immediately immersed in ice-cold isolation buffer. Choroid plexus, meninges, cerebellum, and brain stem were removed, and brains were homogenized. Then, 26% dextran was added, and samples were centrifuged (5800 g; 4 C) for 10 min. The supernatants were discarded; pellets were resuspended and filtered through a 70 μm mesh filter. Filtered homogenates were re-pelleted by centrifugation (1500 g; 10 min) and either smeared on slides for confocal analysis or resuspended in lysis buffer for analysis of protein expression.

### Oxidative stress and glutathione detection

Oxidative stress and intracellular glutathione were detected in brain capillaries using specific fluorescent probes. The main advantages of such an experimental approach are high sensitivity and specificity as well as minimal interference during sample preparation. Freshly isolated “intact” brain microvessels were smeared on glass microscope slides and air-dried before staining. Staining with dihydroethidium (DHE; Molecular Probes/Life Technologies, Grand Island, NY) was used as the indicator of oxidative stress. DHE is membrane permeable and, in the presence of superoxide, is converted to the fluorescent product ethidium bromide, which is trapped by intercalating with DNA [47]. DHE (10 μM in DMSO) was directly applied to brain microvessels and incubated in a light-protected and humidified chamber at 37°C for 90 min. Images were acquired by confocal microscopy.

Monochlorobimane (MCB; Molecular Probes/Life Technologies) was used as a probe for glutathione (GSH) detection. The reaction is based on conjugation of reduced glutathione with MCB [48] with normally nonfluorescent MCB in a reaction catalyzed by glutathione S-transferase. Isolated microvessels were incubated with 100 μM MCB for 90 min at 37°C in cell culture incubator. GSH-MCB fluorescence was detected using a confocal microscope.

### Assessment of tight junction (TJ) proteins

Freshly isolated intact microvessels were spread onto glass microscope slides and heat fixed for 10 min at 95°C. Slides were washed with PBS and fixed in 4% formaldehyde for 10 min at 25°C. Slides were then re-washed with PBS, permeabilized in 0.1% Triton X-100 for 5 min, washed in PBS containing 1% bovine serum albumin (BSA) and then blocked in 1% BSA in PBS for 30 min at 25°C. Slides were incubated overnight at 37°C with appropriate primary antibody (anti-occludin, anti-ZO-1, or anti-claudin-5; all diluted 1:500 in 1% BSA in PBS). The following day, slides were rinsed in 1% BSA and re-blocked with 1% BSA for 30 min. Slides were then incubated with either AlexaFluor 488-conjugated or 546-conjugated IgG (R&D Systems; Minneapolis, MN) for 1 h at 37°C. All slides were washed and mounted with ProLong Gold Antifade reagent (Invitrogen/Life Technologies, Grand Island, NY) containing 4′,6-diamidino-2-phenylindole (DAPI) to visualize the nuclei. Images were acquired using an Olympus BX61WI (Olympus, Center Valley, PA) laser scanning confocal microscope. Acquisition settings for AlexaFlour 488 (excitation at 488 nm and detection range 500–535 nm) and AlexaFlour 546 (excitation 546 nm and detection range 580–620 nm) were visualized as green or red colored fluorescence, respectively. Data were analyzed by Fluoview v. 5 image software (Olympus). Images collected from the treatment and control groups were stained in parallel and collected under uniform instrument setting. Slides prepared in the absence of primary antibody resulted in a loss of specific immunoreactivity.

### Immunoblotting

Lysates of brain microvessels (30 μg protein/lane) were resolved on 10-15% Tris–HCl gels (BioRad) for 60 min at 120 V. The gels were then transferred to 0.45 μm PVDF membranes (Perkin Elmer, Waltham, MA) at 6 V for 20 min and 200 mAmp for 2 h, while immersed in a 10% methanol Tris-glycine (USB, Cleveland, OH) transfer buffer. Membranes were blocked for 1.5 h at 25°C in Superblock blocking buffer (Pierce/Thermo Scientific, Rockford, IL) supplemented with 0.05% Tween 20 before being incubated overnight at 4°C with respective primary antibody diluted in fresh Superblock buffer. Membranes were washed 3 times for 10 min with Tris base saline supplemented with 0.05% Tween 20 (TBST) prior to 2 h incubation with respective secondary antibody diluted in Superblock buffer with 0.05% Tween 20 (1:1,000 for anti-mouse IgG and 1:3,000 for anti-rabbit IgG). Membranes were then re-washed in TBST, developed, and the proteins of interest were detected using the ECL Plus Western blotting detection system (Amersham, Piscataway, NJ). Semi-quantification of protein was performed with NIH Image J software and actin expression was used to normalize the expression results.

### BBB permeability assay

BBB permeability was assessed as described earlier with slight modifications [49]. Mice were injected intraperitoneally (i.p.) with sodium fluorescein (2% in 200 μl PBS) which was allowed to circulate for 30 min. The animals were anesthetized with isofluorane in oxygen, blood was collected via heart puncture, and the mice were perfused with heparinized saline. The brains were harvested and the brain regions (hippocampus, frontal cortex, and putamen) were isolated, immediately immersed in liquid nitrogen and stored at −80°C. The regions were homogenized in PBS (1:10 g/v) followed by protein measurement. The samples were then precipitated in 10% trichloroacetic acid (1:1 v/v) and centrifuged at 1000 g for 10 min. The pH was adjusted by adding 8.33 μl 5 M NaOH to 100 μl supernatant aliquots and fluorescence was detected using a fluorescence plate reader with excitation at 485 nm and emission at 530 nm. BBB permeability was expressed as pg sodium fluorescein/μg protein.

### Statistical analysis

Two-way ANOVA, followed by Student-Newman-Keuls *post hoc* test or two-tailed Student’s *t*-test, was used to compare mean responses among the treatments. A statistical probability of *p* < 0.05 was considered significant.

## Abbreviations

BBB: Blood–brain barrier; BSA: Bovine serum albumin; CNS: Central nervous system; DAPI: 4′, 6-diamidino-2-phenylindole; DHE: Dihydroethidium; GSH: Glutathione; MCB: Monochlorobimane; METH: Methamphetamine; NOX: NAD (P)H oxidase; ROS: Reactive oxygen species; SOD: Superoxide dismutase; TJ: Tight junction; ZO-1: Zonula occludens-1.

## Competing interests

The authors have no conflicting interests to disclose.

## Authors’ contributions

MT designed the studies and wrote the manuscript; MJS, CSR, IEA, LC, and MP performed experiments, KAE, made substantial contribution to conception and design and provided a model of voluntary exercise. All authors read and approved the final manuscript.
